# Engineered and natural gene drives: mechanistically the same, yet not same in kind

**DOI:** 10.1038/s41467-023-41727-3

**Published:** 2023-09-26

**Authors:** Raul F. Medina, Jennifer Kuzma

**Affiliations:** 1 Department of Entomology, Texas A&M University, College Station, TX USA; 2 Genetic Engineering and Society Center, North Carolina State University, Raleigh, NC USA

**Keywords:** Policy, Evolutionary ecology, Synthetic biology

## Abstract

We propose the use of the terms natural gene drive (NGD) and engineered gene drive (EGD) arguing against James et al.^1^, who think both should be included within the term “gene drive”, based on their mechanistic similarities.

Thanks to CRISPR-Cas-based gene editing, engineered gene drive has suddenly become feasible as a potential cost-effective pest control tool that could help us resolve wicked challenges^2,3^. In nature, several organisms harbor genes that “selfishly” drive themselves into populations. This natural gene drive uses similar mechanisms to the ones use today to drive engineered genes into laboratory populations^4^. In this article we disagree with James et al.^1^ who have recently proposed that because natural and engineered gene drives are mechanistically indistinguishable from a molecular standpoint, they should both be referred as “gene drives” because “a gene drive is a gene drive.” We instead propose that two terms be used to distinguish between natural and engineered gene drives, we second Wells and Steinbrecher^5^ arguments, and propose to use the terms *natural gene drive* (NGD) and *engineered gene drive* (EGD).

## EGD as a potential pest control tool

EGD allows humans to push desirable traits into wild populations in super-Mendelian ratios, even if these traits reduce target-organisms’ fitness^2^. In other words, EGD allows us to push driven traits despite their fitness cost to the organisms hosting them. Currently, EGD has worked in mosquitoes and mice, and it shows promise in a variety of other organisms. Although not a single EGD has been released into any shared environment yet, the likelihood of an intentional or accidental release increases with time. Thus, it is important for society to discuss potential EGD releases and their governance now. Because EGD is an emerging technology, most people have not yet familiarized with it, and because most of the current public discussions on this subject refer to public health, even fewer people have any conceptualization of other potential EGD applications.

## Definitions and public trust

To define novel objects, one must deconstruct them. Thus, it is not surprising that novel biotechnology products get conceptually dissected so we can understand how they work, assess their risks and their ethical, legal, and societal implications. However, because biotechnology products do not only exist within scientific realms but also inhabit public arenas, mechanistic characterizations alone are not sufficient to capture their complexities. When developers of emerging technologies use oversimplified product definitions, they reduce public trust and complicate the regulation of these products. Trust is one of the most important factors for public perception of new technologies, and public trust in scientist and regulators will be eventually diminished if we mask the use of genetic engineering through opaque terminology^6^. We believe that it is important to be as nuanced as possible when defining EGD. Oversimplifying any aspect of EGD this early should not occur for there has not been enough time to accumulate sufficient information.

James et al.^1^ propose that NGD and EGD be treated as equivalent by scientists, the public and regulatory bodies. They claim that lumping NGD and EGD under the same term, is necessary to ease the governance of gene drive technologies. We disagree. In fact, we feel that failing to discuss NGD and EGD separately, may negatively impact public trust for the public may perceive this grouping as disingenuous^6^. There is significant evidence from the social science literature that perceptions of novel biotechnology applications are significantly affected by their “naturalness”, with the lack of naturalness as a key reason people reject genetically modified products and other technologies^7–10^. For example, Mielby et al.^8^ find that one public conception of “unnaturalness” is dependent on human interference, and this negatively impacts the public’s perception of genetically modified products. Pooling natural and engineered gene drives together under a common definition could ultimately reduce public trust by increasing the public’s feelings that biotech developers are trying to deceive them by introducing something into the environment that is unnatural (and thus unwanted) under a euphemism.

When a technology is new it is best to keep its elements as deconstructed as possible, lumping them only when data over time have proven different categories to be equivalent. Furthermore, the gene-drive technology development community leans towards technology optimism and may not be the best judge of where the definitional boundaries that affect public perception and risk governance should be set. It is important to bring a wider range of ecology, social science, regulatory, and humanities experts as well as environmental groups and diverse communities, including historically marginalized groups, into conversations about definitions^11^. Definitions affect politics, perceptions, and access to decision-making power, and contribute to the creation of a collective future, that in democratic governances, should be shaped by a plurality of voices.

## Familiar versus uncertain risks

Furthermore, we argue that living organisms are not just their molecular components but also their ecological, evolutionary, and societal complexities. Molecularly identical individuals placed in different ecological communities often affect their ecosystems in significantly different ways. The fact that NGD and EGD are produced by harnessing similar mechanisms does not make their ecological, evolutionary, or societal effects equivalent. One important difference between NGD and EGD is that because organisms hosting naturally driven genes evolved millions of years ago, their impact on natural communities when they first emerged is unknown. However, it is reasonable to assume that the most disruptive ecological impacts of organisms hosting NGD were likely more intense soon after their appearance. Today, organisms hosting naturally driven genes have adapted to their ecosystems and their current ecological risks are familiar. In contrast, the most disruptive impacts of EGD are expected to be witnessed by our species. That is, the ecological risks of deploying organisms hosting EGDs are unrealized yet and therefore currently unknown and unfamiliar. Keeping this in mind, one should expect public risk perception of NGD and EGD to be different. Because public perception can significantly affect the governance of novel technologies, it would be wiser for scientific terms to reflect as much as possible our current level of uncertainty. Terms that better reflect uncertainty are considered more transparent and enjoy higher levels of public trust^12^.

In addition, the types of engineered genes chosen to be driven are likely to differ in kind to the types of genes driven naturally. Mostly, because organisms hosting EGDs will all be benefiting the fitness of our species^5^. Also, if EGD is approved for deployment, it is likely that some communities will deal with not one but several co-existing organisms hosting EGDs. The impact of humans having the ability to choose several engineered genes to be driven in ecological time into natural communities is currently unknown.

Whenever uncertainty is high, granularity and specificity of terms and definitions are always better than coarse conceptualizations. Organisms are not only their molecules but also their interactions with their ecosystems and the effects of these interactions on the biome through time. We believe it is too early to safely define NGD and EGD as the same just because the molecular mechanisms that generate both are similar. The finer scale at which we keep our conceptualizations the better equipped we will be to model their risks and to monitor and remediate any potential harm they may cause.
